# Environmental Context Modulates Habituation of Visually Evoked Defensive Behavior

**DOI:** 10.1523/ENEURO.0434-25.2026

**Published:** 2026-06-02

**Authors:** Haoxuan Qi, Alice Treloar, Greg J. Stuart, Saba Gharaei

**Affiliations:** ^1^Eccles Institute of Neuroscience, John Curtin School of Medical Research, Australian National University, Canberra, ACT 2601, Australia; ^2^Department of Physiology, Neuroscience Program, Biomedicine Discovery Institute, Monash University, Clayton, Victoria 3800, Australia

**Keywords:** environmental context, habituation, innate defensive behavior, mice, sweeping stimuli, visual system

## Abstract

The capacity of animals to rapidly and appropriately respond to potential threats is critical for survival. In many species, this involves innate defensive behaviors, such as flight or freezing. However, not all threats are dangerous. Habituation allows animals to filter out irrelevant stimuli and avoid unnecessary energy expenditure. While environmental context is known to modulate behavior in associative learning paradigms, it remains unclear whether this also applies to visually evoked defensive behaviors. Here, we address this question in mice of either sex by examining the role of environmental context on habituation of defensive responses to threatening visual stimuli. We developed a protocol that produces rapid (within minutes) and stable (lasting at least one week) habituation of freezing responses to slowly sweeping visual stimuli resembling an aerial predator moving across the sky. Using this protocol, we tested the impact of environmental context on habituation and found that changing the context significantly and reversibly reduced the expression of habituation. Specifically, freezing was reinstated when mice were tested in a different context from that used during habituation, reaching levels comparable to nonhabituated animals. Freezing returned to habituated levels when mice were retested in the context used during habituation. In summary, our findings demonstrate that environmental context plays a critical role in shaping habituation of visually evoked innate defensive behaviors. These results reveal a previously unrecognized flexibility in this evolutionarily conserved behavior and highlight the importance of context in regulating responses to visual threats.

## Significance Statement

Animals must balance rapid defensive reactions to potential threats with the need to avoid responding unnecessarily to repeated, nonthreatening events. Habituation supports this balance by reducing defensive responses to repeated, harmless stimuli. Although environmental context is known to shape associative learning, its role in regulating habituation of visually evoked defensive responses has not been directly tested. Here, we show that habituation of innate defensive freezing to a visual threat in mice is strongly context-dependent. Changing to a different context reduces habituation, with habituation restored when mice are returned to the original context. These findings reveal that habituation is not a fixed reduction in responsiveness, but a flexible, context-specific process that enables mice to modify defensive responses to changing environments.

## Introduction

Survival in the natural world requires animals to rapidly and appropriately respond to potential threats (Blanchard and Blanchard, 2008; Tseng et al., 2023; Campos-Cardoso and Cummings, 2025). Across multiple species, this has led to the evolution of innate defensive behaviors that are triggered by biologically relevant threatening stimuli (Fanselow and Lester, 1988; Blanchard and Blanchard, 1989; Kawai et al., 2004; Mancienne et al., 2021; Tseng et al., 2023; Wu and Zhang, 2023). These behaviors are distinct from conditioned fear responses in that they do not depend on prior learning, suggesting that they are mediated by neural circuits designed to detect and react to threats with minimal processing delay (Silva et al., 2016).

The type of defensive behavior expressed depends on the nature of the perceived threat. According to the threat imminence theory, specific sensory cues are matched to specific defensive strategies (Fanselow and Lester, 1988). For example, slowly sweeping visual stimuli, resembling a predator moving across the sky, typically elicit freezing in rodents, presumably to reduce detection (De Franceschi et al., 2016; Solomon et al., 2023). In contrast, looming visual stimuli, which simulate a rapidly approaching aerial predator, usually trigger flight responses (de Vries and Clandinin, 2012; Yilmaz and Meister, 2013; De Franceschi et al., 2016; Solomon et al., 2023). While these behaviors appear stereotyped, emerging evidence suggests that innate defensive behaviors are not fixed and can exhibit a degree of plasticity depending on external conditions and prior experience (Narushima et al., 2022; Carroll et al., 2025; Haimson and Mizrahi, 2025; Mederos et al., 2025).

An important form of plasticity in defensive behaviors is habituation, defined as a progressive decrease in response following repeated stimulus exposure (Thompson and Spencer, 1966; Rankin et al., 2009). Habituation has been extensively characterized across species and response systems, including reflexive, exploratory, and defensive behaviors (Cranney and Ashton, 1985; Thompson and Spencer, 1966; Mackintosh, 1987; Tomsic et al., 1998; Hermitte et al., 1999; Rankin et al., 2009; Jordan et al., 2015). Habituation has an evolutionary advantage in that it enables organisms to filter out irrelevant stimuli and thereby avoid unnecessary energy expenditure (Thompson and Spencer, 1966). With regard to visually evoked defensive behaviors, repeated exposure to both looming and sweeping stimuli can lead to habituation of defensive responses (Tafreshiha et al., 2021; Lenzi et al., 2022; Carroll et al., 2025; Liu et al., 2025; Mederos et al., 2025).

Environmental context plays a critical role in many forms of learning, including fear conditioning (Fanselow, 1980; Phillips and LeDoux, 1992; Bouton, 1993, 2004; Maren et al., 2013). Beyond associative fear conditioning, a substantial literature has examined whether habituation can be context-specific. Empirical studies across species and response systems—including acoustic startle, orienting responses, and exploratory behaviors—have reported that long-term habituation can, under some conditions, depend on the environmental context in which stimulus exposure occurred (Davis and Wagner, 1968; Davis, 1970; Marlin and Miller, 1981; Hall and Channell, 1985; Jordan et al., 2000; Rankin, 2000; Prados et al., 2020; Reyes-Jiménez et al., 2020). However, these effects are not always robust or consistent across paradigms, and recent reviews emphasize that habituation is only sometimes context-specific, depending on factors such as training schedule (Dissegna et al., 2021; Uribe-Bahamonde et al., 2021). Whether contextual modulation extends to visually evoked defensive responses to predator-like stimuli is unknown and is the main focus of this study.

Here, we describe a novel behavioral paradigm that rapidly (within minutes) induces habituation of freezing responses to sweeping visual stimuli in mice. Habituation induced by this paradigm was stable over time (up to at least 1 week). Using this paradigm, we examined the factors that influence the expression and stability of habituation to threatening visual stimuli, finding that changing the environmental context reversibly reduces habituation, whereas returning to the original context restores it. These findings extend established principles of context-modulated habituation to a visually evoked innate defensive behavior and provide a foundation for investigating the neural mechanisms through which context shapes adaptive defensive responses.

## Materials and Methods

### Experimental animals

A total of 26 mice of either sex were used in this study (6–8 weeks of age at the start of the experiment). All mice were housed in a 12:12 reversed light/dark cycle and were acclimatized for 1 week to adjust to this light cycle before the commencement of experiments. Two days prior to experiments, mice were separated into individual cages with enrichment (enrichment roll or wood block). Food and water were provided *ad libitum* throughout the study. All animal procedures were approved by the Animal Experimentation Ethics Committee of the Australian National University.

### Experimental setup

The behavioral arena used in this study was a rectangular opaque box with dimensions of 47 cm in length, 36 cm in width, and 30 cm in height. A Samsung P2050 LCD monitor (38 × 27 cm, 300 cd/m^2^ brightness) was used to present visual stimuli. The monitor was placed above the arena, parallel to the floor. An LED light, mounted on the arena wall, was used to indicate the start and end of visual stimulus presentation, providing a reference point for video analysis. Mice could not see this LED light. Mouse behavior was recorded using a PlayStation 3 Eye Camera at 75 fps in MP4 format. The camera was placed between the LCD monitor and the arena wall, capturing both the LED light and behavioral responses. In addition to a plain, undecorated arena, in some experiments, the arena was decorated with two distinct contexts, which differed visually and tactilely: Context A featured four laminated visual cues (horizontal lines, hollow square dots, vertical lines, and crossed lines) attached to the arena walls using Blu-Tack. Context B had bare walls, but the floor was covered with bubble wrap securely taped to the arena surface.

### Stimulus properties

The sweeping stimulus used during experiments consisted of a 2.5-cm-diameter black disk which traversed the monitor over 4 s from one corner to the opposite corner, moving at a velocity of 11.7 cm/s. Stimuli were generated using Psychtoolbox v3 (Brainard, 1997) in conjunction with the MATLAB R2019 software (MathWorks).

**Figure 1.**
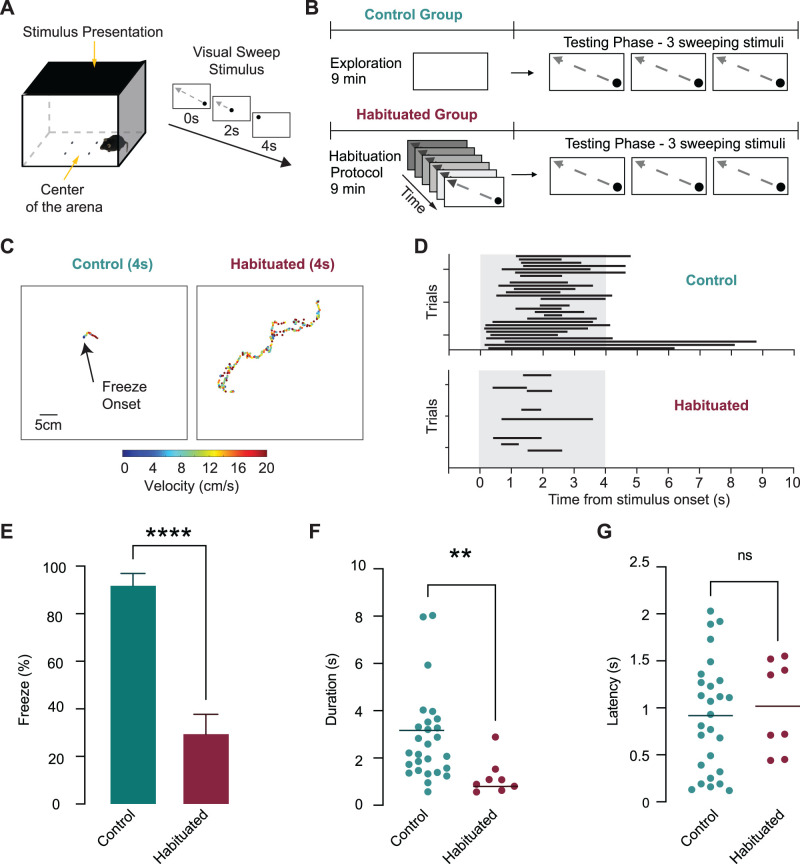
Repeated exposure to sweeping visual stimuli suppresses innate freezing responses. ***A***, Left, Schematic of the testing arena used for the habituation protocol. Right, The visual sweeping stimulus is a small black disk that sweeps diagonally across the overhead monitor over 4 s. ***B***, Experimental design. Mice were randomly assigned to the control group (9 min exploration with no visual stimuli) or the habituated group (130 sweeping visual stimuli increasing in contrast over 9 min). Both groups were subsequently tested with three identical full-contrast sweeping stimuli. ***C***, Representative DeepLabCut trajectory plots showing mouse movement speed and position in the control and habituated groups during the 4 s test sweep stimulus. ***D***, Raster plots showing trial-by-trial freezing responses to the test stimuli in control (teal) and habituated mice (burgundy). Black bars indicate freezing. The shaded gray area indicates stimulus presentation. ***E***, The mean percentage of trials where freezing was observed per mouse (±SEM) for the control (teal) and habituated group (burgundy). Habituated mice froze significantly less than controls. ***F, G***, Freeze duration (***F***) and latency (***G***) in control (teal) and habituated mice (burgundy). Horizontal lines indicate the median. Habituated mice showed significantly shorter duration freezing episodes, but with similar onset latencies.

### Behavioral experiments

Mice in the control group were placed in the arena with the LCD monitor set to a white background and allowed to explore the arena for 9 min. This ensured that control mice had the same amount of time to explore the test arena as the habituated group, minimizing differences due to familiarity with the arena. After this exploration period, control mice were briefly transferred back to their home cage for ∼2 min to avoid exposure to screen flashes during initialization of the Psychtoolbox test sweep program. Once the test sweep program was ready, mice were returned to the arena for testing. The full-contrast test sweep stimulus was manually triggered when mice entered the center of the arena, marked by four dots on the floor (Fig. 1*A*). During each testing session (duration 30 min), mice were presented with up to three sweeping stimuli separated by at least 90 s. Because presentation of sweeping stimuli was only triggered when mice entered the center of the arena, the total number of trials per session and the intertrial interval were not fixed but depended on how often the mouse entered the center of the arena during the testing period. Mice were tested once per day, except when they failed to enter the arena center within 20 min after the start of the testing phase. In these cases, the session was terminated, and the mouse was retested later the same day.

Mice exposed to the habituation protocol were placed in the arena with the LCD monitor initially displaying a dark background. Mice were then exposed to 130 sweeping black disks, which traversed the monitor screen diagonally in 4 s. The protocol began with sweeping black disks presented against a dark background (gray level 2 in MATLAB), with the background incrementally increasing in lightness by two gray levels after each stimulus until the highest contrast condition was reached (gray level 255 in MATLAB), after which the sweeping black disk stimulus was presented against a white background three times at the highest contrast level. Given that each sweep lasted 4 s, the habituation protocol lasted ∼9 min. After the habituation protocol, mice were briefly transferred back to their home cage for ∼2 min during initialization of the Psychtoolbox test sweep program and then returned to the arena for testing using the same full-contrast test sweep stimuli (as explained above).

### Manual and computational analysis of behavioral data

Experiment videos were manually reviewed to identify and classify behavioral responses to the sweeping stimulus. Adobe Premiere Pro 2024 was used to manually determine the onset and offset of freezing behavior by frame-by-frame inspection of the video. Freezing was classified as a sudden reduction in speed, where mouse movement decreased to 2 cm/s or less for a minimum of 0.5 s. Freeze offset was defined as the frame where the mouse resumed movement. During this period, mice must not exhibit any voluntary movements such as grooming, rearing, or sniffing. The absence of these behaviors was crucial for a behavior to be classified as freezing. The analysis window for freezing was constrained to the duration of the stimulus, which was 4 s.

To determine freeze duration, the frame number at which a mouse initially froze was subtracted from the frame number at which the mouse resumed movement. To determine freeze latency, the frame number at the onset of the sweeping stimulus (as detected by LED onset) was subtracted from the frame number at which the mouse initially froze. The difference in frame number was then converted to time in seconds to determine freeze duration and latency based on the frame rate (75 fps). Only mice that exhibited freezing during the test trials were included in duration and latency analyses. DeepLabCut and MATLAB were used in conjunction with manual analysis. Automated analysis using DeepLabCut was primarily used to double-check responses when manual analysis was ambiguous and to plot raster and trajectory figures.

### Statistical tests

All statistical analyses were performed using GraphPad Prism version 10.3.1 (GraphPad Software). For freezing probability, the percentage of freezing trials was first calculated for each mouse and then averaged across mice for statistical comparisons. Data normality was assessed using both the Shapiro–Wilk and D'Agostino–Pearson’s omnibus tests. For comparisons between two groups, unpaired *t* tests were used if data were normally distributed; otherwise, the nonparametric Mann–Whitney test was applied. For comparisons involving more than two groups, one-way ANOVA was used for normally distributed data, followed by Tukey's multiple-comparisons test when significant differences were found. If the data were not normally distributed, the Kruskal–Wallis test was used, followed by Dunn's multiple-comparisons test for post hoc analysis when appropriate. For all analyses, statistical significance was defined as *p* < 0.05. Statistical comparisons are indicated in the figures by asterisks: *p* < 0.05 (*), *p* < 0.01 (**), *p* < 0.001 (***), and *p* < 0.0001 (****). Normally distributed data are reported as mean ± standard error of the mean (SEM), while non-normally distributed data are reported as median with interquartile range (IQR).

## Results

### Rapid habituation to visual sweeping stimulus

We first developed a behavioral habituation protocol to rapidly habituate mice to sweeping visual stimuli. This protocol was based on one recently developed to generate habituation to looming visual stimuli (Lenzi et al., 2022). Mice were placed in an arena with a monitor mounted overhead (Broersen et al., 2025). The monitor was used to display a small black disk sweeping overhead to simulate a cruising aerial predator (Fig. 1*A*). Mice were randomly assigned to a control or habituated group and only tested with the sweeping stimulus whenever they entered the center of the arena (marked by four dots on the floor and manually initiated by an observer). Mice in the control group were allowed to explore the arena for 9 min with no overhead visual stimulus. In contrast, mice in the habituated group were exposed to 130 repetitions of the visual sweeping stimulus over the same 9 min period, during which the screen's background gradually brightened, slowly enhancing the contrast between the sweeping stimulus and the background. Following this 9 min period, we tested the behavioral response of both groups to three full-contrast sweeping stimuli (Fig. 1*B*).

The vast majority of mice in the control group froze in response to the test sweep stimulus, whereas freezing was markedly reduced in the habituated group. In the control group, freezing was observed in 27 out of 29 trials, compared with just 8 out of 31 trials in the habituated group (Fig. 1*C*,*D*; 11 mice per group). The mean percentage of trials where freezing was observed per mouse was significantly reduced in the habituated group compared with control animals (Fig. 1*E*; control, 92.5 ± 5.2%; habituated, 30.1 ± 8.1%; unpaired *t* test, *p* < 0.0001; *n* = 11 mice per group). Habituated mice also froze for significantly shorter durations on the trials in which freezing occurred (Fig. 1*F*; control median, 2.21 s; IQR = 1.47–5.32 s; *n* = 11 mice; habituated median, 0.99 s; IQR = 0.67–1.42 s; *n* = 8 mice; Mann–Whitney test, *p* = 0.0015). The time to freezing onset (freeze latency) did not differ significantly between the two groups (Fig. 1*G*; control, 0.92 s ± 0.11 s; *n* = 11 mice; habituated, 1.02 s ± 0.17 s; *n* = 8 mice; unpaired *t* test, *p* = 0.67). The latency to the first stimulus presentation (defined as the first time the mouse entered the center of the arena) did not differ significantly between control and habituated mice (control, 134.3 ± 46.2 s; habituated, 158.4 ± 75.0 s; unpaired *t* test, *p* = 0.79). This indicates that exploratory behavior and initial threat sensitivity were comparable between the two groups. Together, these results demonstrate that gradual exposure to a sweeping visual stimulus can rapidly (within minutes) induce habituation of innate freezing responses in mice, reflected by reduced frequency and duration of freezing behavior.

### Habituation memory lasts for at least 1 week

**Figure 2.**
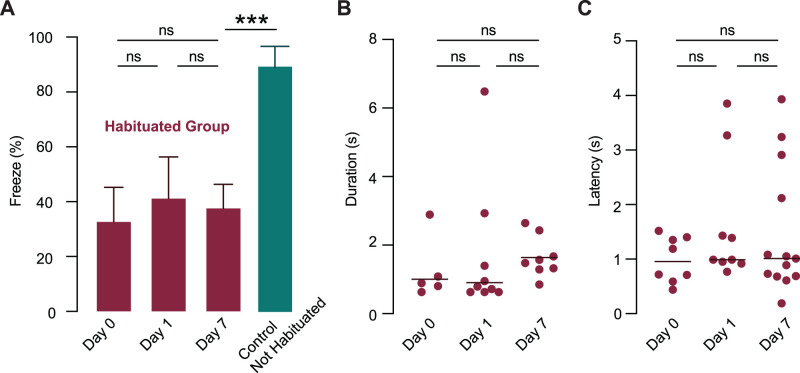
Habituation to sweeping visual stimuli is stable for at least a week. ***A***, The mean percentage of trials where freezing was observed per mouse (±SEM) in habituated mice (burgundy) immediately after the habituation protocol (Day 0), 24 h later (Day 1), and 1 week later (Day 7) compared with nonhabituated controls (teal). No significant differences were observed across days. ***B, C***, Freeze duration (***B***) and latency (***C***) on the different testing days. Horizontal lines indicate the median. No significant differences were found across days.

To assess the stability of habituation, mice were tested at three time points: on the same day as the habituation protocol (Day 0), 24 h later (Day 1), and 1 week later (Day 7). Freezing was observed in 5 of 19 trials on Day 0, 9 of 22 trials on Day 1, and 8 of 21 trials on Day 7. Correspondingly, the mean percentage of trials where freezing was observed per mouse was low at all three time points, with no significant difference in the extent of freezing across the days tested (Fig. 2*A*; Day 0, 33.1 ± 12.7%; *n* = 7 mice; Day 1, 41.5 ± 15.1%; *n* = 8 mice; Day 7, 38.0 ± 8.8%; *n* = 7 mice; one-way ANOVA, *F*_(2,19)_ = 0.1126; *p* = 0.8941). As expected, nonhabituated control mice exhibited significantly higher freezing than habituated mice at all time points (Fig. 2*A*; one-way ANOVA including control group, *p* = 0.0004). Similarly, freeze duration did not differ significantly between any of the test days (Fig. 2*B*; Day 0 median, 0.95 s; IQR = 0.72–1.10 s; *n* = 5 mice; Day 1 median, 0.79 s; IQR = 0.63–2.17 s; *n* = 5 mice; Day 7 median, 1.29 s; IQR = 0.71–1.62 s; *n* = 6 mice; Kruskal–Wallis test, *H* = 0.3551; *p* = 0.8373; *n* = 16 mice). Freeze latency also remained stable over time, with no significant difference observed between test days (Fig. 2*C*; Day 0 median, 0.96 s; IQR = 0.62–1.39 s; *n* = 5 mice; Day 1 median, 0.99 s; IQR = 0.94–2.35 s; *n* = 5 mice; Day 7, 1.01 s; IQR = 0.69–2.52 s; *n* = 6 mice; Kruskal–Wallis test, *H* = 1.557; *p* = 0.4591; *n* = 16 mice). Together, these results indicate that habituation to sweeping visual stimuli is robust and persists for at least 1 week following a single habituation session.

### Habituation to sweeping stimuli generalizes across stimulus direction

**Figure 3.**
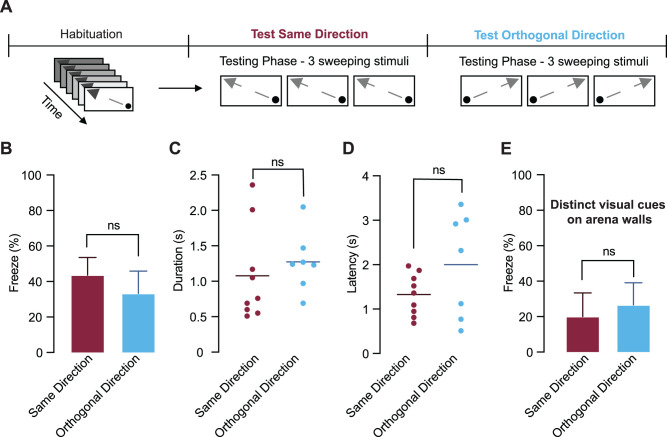
Habituation to sweeping visual stimuli does not depend on stimulus direction. ***A***, Schematic of the experimental design. After habituation, mice were tested with three sweeping stimuli either in the same direction as during habituation or in an orthogonal direction. ***B***, The mean percentage of trials where freezing was observed per mouse (±SEM) when mice were tested with a sweeping stimulus presented in the same (burgundy) or the orthogonal (blue) direction relative to that presented during the habituation protocol. No significant differences were observed between groups. ***C, D***, Freeze duration (***C***) and latency (***D***) when mice were tested with a sweeping stimulus presented in the same (burgundy) or the orthogonal direction (blue) to that used during habituation. Horizontal lines indicate the median. Freezing duration and latency did not depend on stimulus direction. ***E***, The mean percentage of trials where freezing was observed per mouse (±SEM) when distinct visual cues were added to the arena. Mice were tested with a sweeping stimulus presented in the same (burgundy) or orthogonal (blue) direction relative to that used during habituation (*n* = 5 mice per group). No significant differences were observed between groups.

To test whether habituation was specific to the direction of the habituating sweeping stimulus, mice were habituated to a sweeping stimulus in one direction and then tested using a sweeping stimulus that was either in the same or the orthogonal direction (Fig. 3*A*). Freezing was observed in 9 of 21 trials when the direction of the test sweep stimulus was the same as during the habituation protocol, compared with 7 of 23 trials when the direction of the test sweep stimulus was orthogonal to that during the habituation protocol. The mean percentage of trials where freezing was observed per mouse did not differ significantly between these two test groups (Fig. 3*B*; same direction, 43.6 ± 9.9%; *n* = 8 mice; orthogonal direction, 33.3 ± 2.6%; *n* = 8 mice; unpaired *t* test, *p* = 0.5289). Similarly, no significant difference was observed in freeze duration (Fig. 3*C*; same direction, 1.08 ± 0.22 s; *n* = 7 mice; orthogonal direction, 1.27 ± 0.16 s; *n* = 5 mice; unpaired *t* test, *p* = 0.511) or in freeze latency (Fig. 3*D*; same direction, 1.33 ± 0.16 s; *n* = 7 mice; orthogonal direction, 2 ± 0.45 s; *n* = 5 mice; unpaired *t* test, *p* = 0.1371). In supplementary experiments (10 mice), distinct visual cues were added to each wall of the arena to facilitate spatial orientation; however, this manipulation did not alter freezing probability or produce direction-specific freezing responses to the sweeping stimulus (Fig. 3*E*; same direction, 20 ± 13.38%; *n* = 5 mice; orthogonal direction, 26.6 ± 12.51%; *n* = 5 mice; unpaired *t* test, *p* = 0.7179). These results indicate that habituation to sweeping stimuli does not depend on the direction of the sweeping stimulus.

### Habituation to sweeping stimuli is context-specific

**Figure 4.**
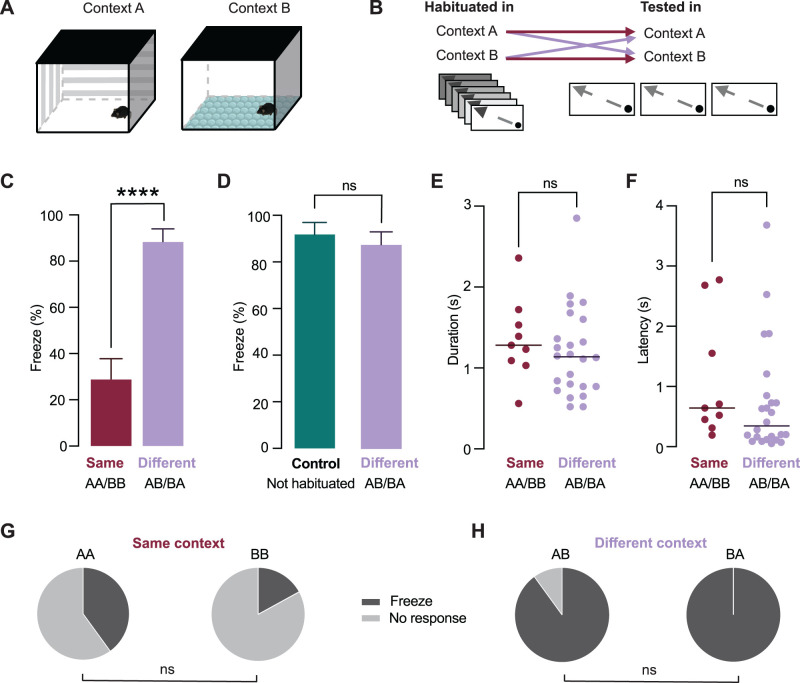
Habituation to sweeping visual stimuli is context-specific. ***A***, Schematic illustration of the two environmental contexts used during the experiment. Context A contained distinct visual cues on the arena walls, while Context B lacked wall cues and included bubble wrap on the floor to provide additional tactile input. ***B***, Experimental design. Mice were first habituated to the sweeping visual stimulus in one context (Context A or Context B) and were subsequently tested in either the same (AA/BB) or a different context (AB/BA). ***C***, The mean percentage of trials where freezing occurred per mouse (±SEM) during the three test presentations of the sweeping stimulus. Mice tested in the different context (purple) showed significantly more freezing than mice tested in the same context (burgundy), indicating reduced expression of habituation. ***D***, Comparison between naive control mice and habituated mice tested in a different context. The percentage of freezing trials did not differ between groups, indicating that testing in a different context restored freezing responses to levels comparable to naive animals. ***E, F***, Freeze duration (***E***) and latency (***F***) when mice were tested in the same (burgundy) or in a different context (purple) from that used during habituation. Horizontal lines indicate the median. There was no significant difference between mice tested in the same or a different context. ***G***, Pie charts showing there was no significant difference in the proportion of freezing trials in mice habituated in Context A and tested in Context A, compared with mice habituated in Context B and tested in Context B. ***H***, Pie charts showing there was no significant difference in the proportion of freezing trials in mice habituated in Context A and tested in Context B, compared with mice habituated in Context B and tested in Context A.

We next tested whether habituation of innate defensive responses to sweeping visual stimuli depends on the environmental context. To test this, mice were habituated in one of two distinct contexts: Context A, which featured unique visual cues on the arena wall, and Context B, which had no visual cues on the arena walls and bubble wrap on the floor to provide tactile input (Fig. 4*A*). Following habituation, mice were tested in either the same context in which they were habituated or in a different context (Fig. 4*B*). The direction of the visual sweeping stimuli used for testing was identical in both groups. Freezing was observed in 8 of 27 trials when mice were tested in the same context as used during habituation and in 24 of 27 trials when mice were tested in a different context. The mean percentage of trials where freezing was observed per mouse was significantly higher when mice were tested in a different context from that used during habituation compared with when they were tested in the same context (Fig. 4*C*; same context, 29.6 ± 8.7%; *n* = 9 mice; different context, 89.0 ± 5.5%; *n* = 9 mice; unpaired *t* test, *p* < 0.0001). To determine whether the elevated freezing observed in the different-context condition resembled responses in naive animals, we compared these mice with nonhabituated control animals. The mean percentage of trials where freezing was observed per mouse did not differ between control mice and mice tested in a different context from that used during habituation (Fig. 4*D*; control, 92.5 ± 5.2%; different context, 89.0 ± 5.5%; unpaired *t* test, *p* = 0.65; *n* = 11 control mice; *n* = 9 different-context mice). Despite the difference in freezing between mice tested in the same and different contexts from that used during habituation, there was no difference in freeze duration (Fig. 4*E*, same context median, 1.28 s; IQR = 1.06–1.63 s; *n* = 6 mice; different context median, 1.14 s; IQR = 0.77–1.54 s; *n* = 9 mice; Mann–Whitney test, *p* = 0.375) or latency (Fig. 4*F*; same context median, 0.64 s; IQR = 0.38–2.12 s; *n* = 6 mice; different context median, 0.35 s; IQR = 0.13–0.82 s; *n* = 9 mice; Mann–Whitney test, *p* = 0.1507). The latency to the first stimulus presentation also did not differ significantly between mice tested in the same or different context from that used during habituation (same, 120.5 ± 24.4 s; different, 137.4 ± 35.6 s; unpaired *t* test, *p* = 0.7), indicating similar exploratory behavior in these two populations. The overall duration of the testing sessions was also similar between mice tested in the same or different context from that used during habituation (same context, 8.61 ± 2.80 min; different context, 8.12 ± 2.79 min), indicating that the time interval between sweeping test stimuli was, on average, similar. These data indicate that the observed difference in freezing between these two groups was not due to differences in exploratory behavior, stimulus exposure, or general arousal, but instead reflects a context-dependent modulation of habituated defensive responses.

To test whether this context-specific effect was due to intrinsic differences in threat salience, we separated the data based on which context was used for habituation. There was no significant difference in the proportion of freezing trials when mice were habituated in Context A compared with Context B (Fig. 4*G*; Context A, 33%, *n* = 6 trials; Context B, 17% freezing, *n* = 6 trials; Fisher's exact test, *p* > 0.99). Similarly, when mice were tested in a context different from that used during habituation the proportion of freezing trials did not depend on whether mice were habituated in Context A or Context B (Fig. 4*H*; Context A to B, 83% freezing, *n* = 6 trials; Context B to A, 100% freezing, *n* = 6 trials; Fisher's exact test, *p* > 0.99). Together, these results indicate that habituation to sweeping visual stimuli is context-specific, demonstrating that contextual cues play a crucial role in gating the habituated state.

### Context-specific habituation is reversible

**Figure 5.**
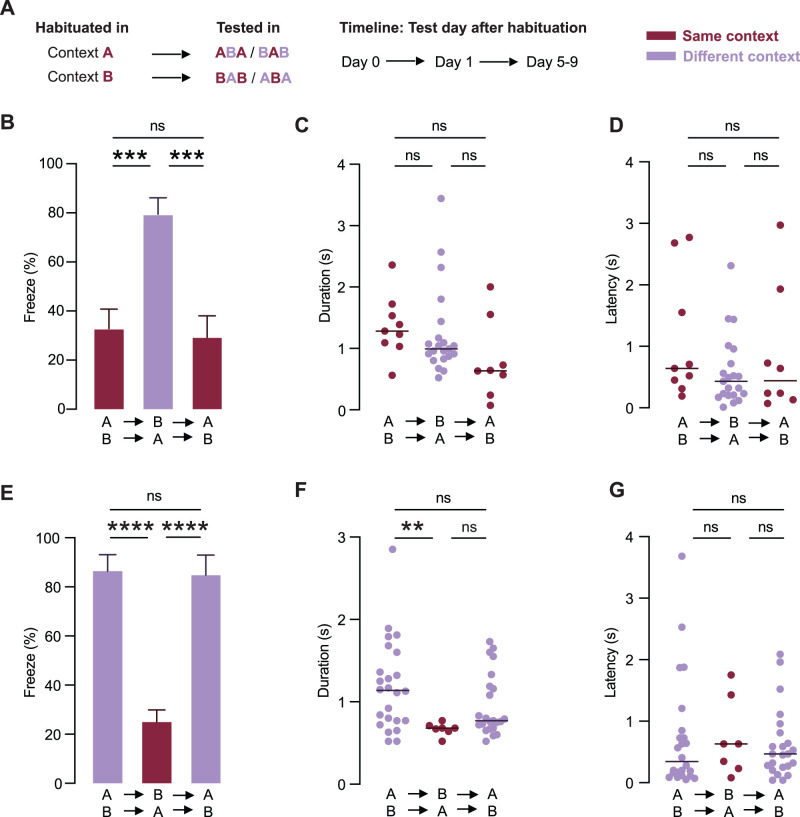
Context-specific habituation to sweeping visual stimuli is reversible. ***A***, Experimental design for the context-reversal paradigm. Mice were habituated in either Context A or B and tested across three conditions: same or different context (Day 0), context reversed (Day 1), and returned to the original context (Day 5–9). ***B***, The mean percentage of trials where freezing was observed per mouse (±SEM) across testing conditions. Mice were initially tested in the same context as that used during habituation (Day 0; burgundy), then tested in a different context (Day 1; purple), and then retested in the context used during habituation (Day 5–9; burgundy). ***C, D***, Freeze duration (***C***) and latency (***D***) in mice initially tested in the same context as used during habituation (Day 0; burgundy), then tested in a different context (Day 1; purple), and then returned to the original context (Day 5–9; burgundy). Horizontal lines indicate the median. There was no significant difference between testing days. ***E***, The mean percentage of trials where freezing was observed per mouse (±SEM) across testing conditions. Mice were initially tested in a different context from that used during habituation (Day 0, burgundy), then tested in the habituated (same) context (Day 1, purple), and then retested in the different context from that used during habituation (Day 5–9, burgundy). Horizontal lines indicate the median. There was a significant difference between testing days. ***F, G***, Freeze duration (***F***) and latency (***G***) in mice initially tested in a different context (Day 0, burgundy), then tested in the habituated (same) context (Day 1, purple), and then retested in the different context (Day 5–9, burgundy). Horizontal lines indicate the median.

While the previous experiments showed that habituation depends on context, it is unclear whether this reflected a general suppression of freezing in the new context or whether habituation was specifically linked to the context in which training occurred. To address this, we performed a context-reversal experiment to determine whether returning animals to the same context in which they were habituated would restore the expression of habituation (Fig. 5*A*). Mice were first habituated in either Context A or Context B. They were then tested in either the same or a different context (Day 0). The following day (Day 1) the test context was reversed, and then later that week (Day 5–9) mice were retested in the context used on Day 0. This design allowed us to determine whether habituation is both context-specific and reversible.

These experiments (Fig. 5*B*) revealed that freezing was low when mice were tested in the same context as they were exposed to during habituation (Day 0; 33.2 ± 7.9%; 9 of 27 trials; *n* = 9 mice) but significantly increased when mice were tested in a different context the next day (Day 1; 79.8 ± 6.6%; 21 of 25 trials; *n* = 9 mice), consistent with restoration of defensive responses in the new context as described in Figure 4. Importantly, freezing returned to low levels when mice were retested in the same context used during habituation (Day 5–9; 29.6 ± 8.7%; 7 of 26 trials; *n* = 9 mice; one-way ANOVA, *F*_(2,24)_ = 12.89; *p* = 0.0002). Despite this context-specific reversal in habituation, freeze duration and latency did not significantly differ between the different contexts (Fig. 5*C*,*D*; Kruskal–Wallis test, *H* = 5.744; *p* = 0.0566 for freeze duration; *H* = 2.231; *p* = 0.3277 for freeze latency).

In complementary experiments (Fig. 5*E*), we found that freezing was high when mice were first tested in the different context (Day 0; 87.1 ± 6.6%; 24 of 26 trials; *n* = 9 mice) but decreased to low levels when tested in the habituated context (Day 1; 25.7 ± 4.9%; 7 of 26 trials; *n* = 9 mice). Importantly, freezing returned to high levels when mice were retested in the different context from that used during habituation (Day 5–9; 85.2 ± 8.1%; 23 of 27 trials; *n* = 9 mice; one-way ANOVA, *F*_(2,24)_ = 27.53; *p* < 0.0001). Freeze duration was significantly lower in these mice when they were tested in the same context as that used during habituation (Fig. 5*F*; Kruskal–Wallis test, *H* = 9.275; *p* = 0.0097; Dunn's post hoc test, Day 0 vs Day 1, *p* = 0.0084), whereas freeze latency was not dependent on context (Fig. 5*G*; Kruskal–Wallis test, *H* = 0.3702; *p* = 0.8308). Together, these results demonstrate that the expression of habituation is gated by environmental context and can be reversibly modulated by changes in context.

## Discussion

In this study, we developed a behavioral protocol that rapidly induces habituation of freezing responses to slowly sweeping visual stimuli in mice. Consistent with recent work using looming stimuli, we show that it is possible to rapidly habituate mice to slowly sweeping visual stimuli within minutes (Lenzi et al., 2022; Mederos et al., 2025). Using this paradigm, we found that environmental context plays a critical role in determining the impact of habituation on visually evoked defensive behaviors. Specifically, we found that changing the context significantly and reversibly reduced the expression of habituation of freezing responses to slowly sweeping stimuli resembling an aerial predator cruising overhead. These findings demonstrate that habituation of visually evoked defensive behaviors is flexible and dependent on the specific environmental context. One interpretation of these findings is that contextual cues influence the expression of previously habituated responses. Our findings extend previous demonstrations of context-specific habituation to an ethologically relevant visual threat paradigm.

Looming and overhead sweeping visual stimuli reliably evoke defensive responses in naive mice, reflecting activation of evolutionarily conserved visual threat-detection mechanisms. Importantly, although these responses are termed innate, our findings demonstrate that their expression is flexible and shaped by experience and environmental context. Evidence for context-dependent habituation has previously been reported during acoustic startle, orienting responses, and exploratory behaviors (Davis, 1970; Marlin and Miller, 1981; Jordan et al., 2000; Rankin, 2000; Prados et al., 2020; Reyes-Jiménez et al., 2020). However, these effects are not uniformly observed, and recent reviews emphasize that the context dependence of habituation varies depending on factors such as the amount and spacing of training, the time between training and testing, and the specific behavioral paradigm used (Dissegna et al., 2021; Uribe-Bahamonde et al., 2021). Our findings align with this broader literature by demonstrating that long-term habituation of visually evoked freezing behaviors can be modulated by context under our testing conditions.

We induced robust habituation to sweeping visual stimuli within minutes. Our habituation protocol was based on a recent study (Lenzi et al., 2022) that achieved rapid suppression of flight responses to a looming visual stimulus by gradually increasing the stimulus-background contrast across repeated presentations. This work highlights how changes in stimulus salience can drive plasticity of innate defensive circuits. We adapted this “contrast-ramping” approach to sweeping stimuli, which elicit freezing rather than flight, and observed a similar rapid reduction in defensive behavior. This finding indicates that the same manipulation can be applied across different types of visual threat stimuli and defensive responses, suggesting that gradual contrast enhancement may be a broadly effective strategy for attenuating innate defensive behaviors. One possibility is that a progressive increase in visual salience allows time for perceptual reevaluation and thus diminishes the perceived threat level, potentially facilitating reclassification of the stimulus as nonthreatening. Alternatively, contrast ramping may engage neural circuits involved in sensory prediction or top–down inhibition, thereby reducing activation of defense pathways (Wei et al., 2015; Baier et al., 2025). Together, these findings demonstrate that dynamic manipulation of sensory input can serve as a powerful tool for probing and shaping innate behavioral responses.

Previous studies on innate defensive responses suggest that these responses are tightly conserved, species-specific, and evolutionarily tuned to specific ecological threats (Blanchard and Blanchard, 1971; Bolles, 1971; Dielenberg and McGregor, 2001; Mancienne et al., 2021; Baier et al., 2025). According to this view, the specific behavior elicited depends on the stimulus conditions, with different behaviors triggered by overhead movement, looming expansion, or predator-like silhouettes, each presumably generated by hard-wired neural circuits designed to ensure survival. Consistent with this idea, earlier work has shown that habituation of innate defensive responses depends on critical stimulus parameters, such as the surface area and shape of the threatening visual stimulus, the contrast between the stimulus and background, and stimulus speed, with increasing speed reinstating defensive behavior even after habituation (Yilmaz and Meister, 2013; Tafreshiha et al., 2021). Based on these observations, one might expect that changing the direction of a sweeping stimulus would also disrupt habituation. In contrast, our results reveal that habituation to sweeping visual stimuli is not dependent on stimulus direction. Specifically, mice that were habituated to sweeping stimuli in one direction displayed comparable reductions in freezing when tested with stimuli moving in the orthogonal direction. The absence of a dependence of habituation on stimulus direction may indicate that neural representations encode broader classes of visual threat stimuli that generalize across changes in stimulus direction. Alternatively, as mice freely move in the arena during the habituation session, they will be exposed to the habituating stimulus from multiple directions. This may diminish the salience of direction as a distinguishing factor, explaining why changing stimulus direction does not restore defensive behaviors.

Theoretical accounts differ in how habituation and its contextual modulation are generated. Dual-process theory proposes that habituation and sensitization operate in parallel and may be differentially influenced by stimulus intensity and arousal variables (Thompson and Spencer, 1966; Groves and Thompson, 1970) but does not explicitly predict context-specific long–term habituation. Similarly, stimulus–model comparator theories emphasize the formation of internal representations against which incoming stimuli are compared (Sokolov, 1963) yet do not formally incorporate contextual retrieval as a determinant of the recovery of behavioral responses following habituation. In contrast, Wagner's model proposes associations between the environmental context and the repeated stimulus (i.e., the context becomes predictive of the stimulus based on prior experience). In this framework, repeated stimulus exposure activates a representation in short-term memory (STM), reducing responding via self-generated priming, a process in which repeated presentation of a stimulus activates an internal representation of that stimulus in STM, thereby reducing the behavioral response to subsequent presentations. As a result, the stimulus effectively predicts its own occurrence, leading to a transient suppression of responding. Over longer intervals, associations may form between the stimulus and contextual cues and be stored in long-term memory. When the stimulus is later encountered in the same context, contextual cues can reactivate the stored representation of the stimulus in STM, thereby maintaining the reduced behavioral response. In a novel context, where such cues are absent, this reactivation does not occur, and the behavioral response can return toward its prehabituation level (Wagner, 1981; Uribe-Bahamonde et al., 2019; Uribe-Bahamonde et al., 2021). The present findings are compatible with this retrieval-based theory, insofar as contextual changes modulated the expression of long-term habituation. However, the present behavioral data do not allow us to determine whether the contextual modulation observed in our experiments reflects an associative stimulus–context retrieval process, nonassociative memory dynamics, or an interaction between the two. Accordingly, our results are compatible with, but do not provide support for, retrieval-based theories of context-specific habituation. Similar context-dependent retrieval processes have been extensively characterized in associative learning paradigms such as extinction and latent inhibition, where phenomena including renewal and reinstatement reveal how contextual cues regulate the expression of learned behavior (Bouton, 2004; Maren et al., 2013).

Future work could further dissect the nature of contextual control of visually evoked defensive behaviors using experimental designs adapted from the contextual learning literature (Bouton and Bolles, 1979; Bouton and King, 1983; Hall and Channell, 1985; Westbrook et al., 2000, 2002; Bouton, 2004; Westbrook and Bouton, 2010; Maren et al., 2013). For example, familiar-context shift paradigms could be used, in which animals are first exposed to a context without the visual stimulus, then habituated to the stimulus in a different context, and finally tested in the initially experienced context. In this design, the test context is familiar but was never associated with the stimulus, allowing dissociation between contextual familiarity and context–stimulus learning. Extinction-like manipulations could also be employed, in which animals are reexposed to the habituation context in the absence of the stimulus prior to testing, to determine whether context-alone exposure modifies the expression of habituation. In addition, latent inhibition-like designs could be used, in which animals are first preexposed to a context without the stimulus before habituation occurs in that same context, to assess whether prior context exposure alters the strength of habituation. Together, these paradigms would allow more precise determination of whether habituation of visually evoked defensive behaviors depends on learned associations between the stimulus and environmental context and whether its expression reflects context-dependent retrieval processes like those described in other learning systems.

Together, these findings indicate that habituation of visually evoked defensive behavior cannot be fully explained as a purely stimulus-driven process. Instead, repeated stimulus exposure appears to interact with learned properties of the environment, such that contextual cues modulate the expression of defensive responses. This interpretation links classical accounts of habituation with associative learning frameworks and suggests that even behaviors traditionally considered nonassociative may be shaped by contextual information under ethologically relevant conditions.

While the superior colliculus (SC) is known to be involved in the rapid detection of threatening visual stimuli and plays a central role in initiating innate defensive responses such as freezing and flight (Shang et al., 2018), the context dependence of habituation likely involves higher-order brain regions that mediate contextual learning and emotional regulation. In particular, the hippocampus and the amygdala have been implicated in encoding contextual associations and orchestrating defensive behaviors (Josselyn et al., 2015; Kim and Cho, 2020; Terranova et al., 2022). The amygdala is known as a key structure for initiating freezing responses and receives threat-related visual input via excitatory projections from the SC through the lateral posterior thalamic nucleus (Wei et al., 2015). The hippocampus, in contrast, encodes contextual information and exerts top–down control of amygdala activity through direct projections (Josselyn et al., 2015; Kim and Cho, 2020; Terranova et al., 2022). Previous work has shown that the strength of the hippocampal projection to the basolateral amygdala (BLA), particularly from the ventral CA1, increases during threat learning, enabling context-specific retrieval of aversive memories (Jimenez et al., 2020; Kim and Cho, 2020). In addition, reactivation of hippocampal engram cells active in a context that was previously associated with threat can drive the activation of amygdala engram neurons, allowing the animal to express defensive behaviors (Josselyn et al., 2015). Hippocampal contextual engrams can also flexibly encode memories of different valence, suggesting that hippocampal representations may influence whether defensive or safety-related responses are expressed (Redondo et al., 2014). Other studies have shown that the BLA contains distinct neuronal populations encoding positive and negative valence, which can exert mutual inhibitory control over one another (Beyeler et al., 2016; Kim et al., 2016). Repeated exposure to a previously threatening stimulus without adverse consequences may recruit safety-related ensembles within the BLA that suppress activity in fear-encoding neurons. Such a mechanism could account for the context dependence of habituation observed here, in which repeated exposure to the sweeping stimulus reduces freezing, while a change in environmental context reinstates defensive behaviors. Although habituation involves a reduction rather than an enhancement of fear responses, these findings suggest that hippocampal–amygdala circuits may link contextual information with the regulation of defensive responses. One possibility is that contextual cues engage hippocampal–amygdala pathways that bias activity toward safety-encoding circuits in the BLA, leading to expression of habituated defensive responses. Future circuit-level investigations will be required to directly test this hypothesis.

In humans, contextual modulation plays a critical role in shaping fear responses, and its disruption is a hallmark of anxiety-related disorders. In post-traumatic stress disorder (PTSD), for example, environmental cues associated with prior trauma can evoke intense defensive reactions even in the absence of a direct threat (Liberzon and Sripada, 2008). These maladaptive responses are thought to arise from impaired contextual discrimination, where fear is not appropriately suppressed in safe contexts (Grillon, 2002). Neuroimaging studies have consistently shown hyperactivity of the amygdala and altered hippocampal function in individuals with PTSD, reflecting a breakdown in the neural circuits involved in contextual fear regulation (Liberzon et al., 1999; Tsoory et al., 2007; Shin and Liberzon, 2010). While habituation in humans is less well understood, studies show that defensive responses can reappear in novel or trauma-associated contexts, suggesting that, as in rodents, contextual cues play a critical role in shaping behavior (LaBar and Phelps, 2005; Andreatta and Pauli, 2021). Together, these observations highlight context as a key factor in modulating both innate and learned fear responses in both rodents and humans.

In conclusion, our findings establish a rapid and robust paradigm for habituating innate defensive behaviors in mice. Using this paradigm, we demonstrated that habituation is modulated by environmental context. These observations highlight flexibility in defensive responses, which are often considered hard-wired. Our study also presents a tractable model for examining how threat responses are dynamically influenced by experience. Given that deficits in habituation have been linked to neuropsychiatric conditions such as PTSD, autism spectrum disorder, and attention-deficit/hyperactivity disorder (McDiarmid et al., 2017; Jamal et al., 2021), understanding how the brain flexibly suppresses defensive behaviors in safe environments may inform future investigations into maladaptive fear and hypervigilance in these disorders. Future studies investigating habituation and its context dependence at the cellular and circuit level will increase our understanding of how neural systems integrate threats with experience-dependent signals of safety, enabling animals to flexibly regulate defensive behaviors in changing environments.

## Data availability

The data that support the findings of this study are available from the corresponding authors upon reasonable request.

## Synthesis

Reviewing Editor: Mark Sheffield, University of Chicago Division of the Biological Sciences Decisions are customarily a result of the Reviewing Editor and the peer reviewers coming together and discussing their recommendations until a consensus is reached. When revisions are invited, a fact-based synthesis statement explaining their decision and outlining what is needed to prepare a revision will be listed below. The following reviewer(s) agreed to reveal their identity: Michael Fanselow. Note: If this manuscript was transferred from JNeurosci and a decision was made to accept the manuscript without peer review, a synthesis may not be available.

The main issue raised is that the paper overstates novelty and certainty. Several statements imply this is the first demonstration that habituation of an innate defensive response is context-specific, and that the context-dependence of habituation in general is unknown. Reviewers argue these claims are not supported given prior empirical and review literature documenting context-specific habituation (including work on acoustic startle and broader syntheses of context-specific habituation). The consensus is that the manuscript should more accurately position the contribution as extending context-specific habituation to this visual defensive assay (and under these conditions), rather than presenting the phenomenon as newly discovered. Importantly, the reviewers' concern is not that incremental work is unpublishable, but that the manuscript must be rigorous, accurate, and properly contextualized with appropriate citation and careful language.

A second, closely related theme is that the interpretation relies at times on intuitive but insufficiently supported logic, especially around the use of terms like "innate" and "defensive." Reviewers recommend greater conceptual precision: distinguishing whether the motor pattern is unlearned from whether the stimulus meaning (i.e., being innately perceived as dangerous) is hard-wired, and avoiding strong claims about innateness without the kind of empirical analysis that would justify them. This concern extends to how the authors differentiate their findings from associative learning frameworks. One reviewer notes that existing theory (which the authors already cite) explicitly treats long-term habituation as context-linked learning, and suggests that the results may also be interpretable through alternative associative accounts (e.g., contextual conditioning, extinction-like processes, and renewal). As written, the manuscript is seen as too confident that the findings reflect a non-associative mechanism that is conceptually distinct from contextual fear learning; reviewers encourage a more balanced discussion that acknowledges plausible competing interpretations and clarifies what the current data can and cannot resolve.

There are also methodological and reporting issues that reviewers feel must be addressed to ensure rigor and interpretability. Concerns include potential confounds introduced by presenting stimuli only when animals occupy a particular location (which may correlate with anxiety state and reduces experimenter control over trial timing and trial counts), raising questions about balancing and statistical comparability across subjects. Reviewers also flag that the operational definition of freezing appears unusually liberal compared with standard usage in the field, which could affect interpretation and cross-study comparability. In addition, at least one key figure and its timeline/logic are described as confusing, limiting the reader's ability to determine whether the most informative design (e.g., a clearly articulated A-B-A test of retention after exposure to a new context) is actually implemented and what the results show.

Finally, reviewers suggest several concrete improvements that would strengthen the empirical presentation without necessarily expanding the experimental scope: clarifying the experimental schematic and results presentation for the context-switch experiment; adding appropriate control groups (e.g., non-habituated controls) to support key comparisons; correcting citation details; and substantially revising the Introduction and Discussion to accurately reflect the state of the literature, appropriately calibrate claims, and present a more careful, theory-aware interpretation.
